# Preparation and Properties of Sulfonated Poly(phthalazinone ether ketone) Membranes for Electrodialysis

**DOI:** 10.3390/polym14091723

**Published:** 2022-04-23

**Authors:** Cong Deng, Qian Liu, Shouhai Zhang, Zhaoqi Wang, Yuning Chen, Xigao Jian

**Affiliations:** 1 State Key Laboratory of Fine Chemicals, School of Chemical Engineering, Dalian University of Technology, Dalian 116024, China; flutdeng556@gmail.com (C.D.); cavenlouis@outlook.com (Q.L.); kisekiwang@outlook.com (Z.W.); yuning-chen@outlook.com (Y.C.); jian4616@dlut.edu.cn (X.J.); 2 Dalian Key Laboratory of Membrane Materials and Membrane Processes, High Performance Polymer Engineering Research Center, Dalian 116024, China

**Keywords:** sulfonated poly(aryl ether ketone)s, cation exchange membranes, electrodialysis, concentration, desalination

## Abstract

Sulfonated poly(phthalazinone ether ketones) (SPPEK) with ion exchange capacities from 0.77 to 1.82 mmol·g^−1^ are synthesized via an electrophilic substitution reaction. Nuclear magnetic resonance and infrared absorption spectroscopy are used to characterize the chemical structure of the obtained polymers for confirming the successful introduction of sulfonic groups. SPPEKs show excellent thermal stability; their temperature required to achieve 5% weight loss is about 360 °C. Accordingly, the obtained membranes possess high ion perm-selectivity, proton conductivity, and low area resistance. Regarding the electrodialysis-related performance of the membranes, the SPPEK-4 membrane has the highest limiting current density (39.8 mA·cm^2^), resulting from its high content of sulfonic groups. In a desalination test of standard solution, SPPEK-3 and SPPEK-4 membranes exhibit both better salt removal rate and acceptable energy consumption than commercial membrane. Additionally, SPPEK-3 membrane shows outstanding performance in terms of high concentration rate and low energy consumption during saline water treatment, which indicates the feasibility of novel membranes in electrodialysis application.

## 1. Introduction

The electrodialysis (ED) process is the selective transport of ion to treatment feeding streams under applied electric fields [1]. The ED process has been applied in several fields, including food production, wastewater treatment, acid recovery, and desalination, due to its low cost, easy maintenance, and convenient operation [2,3,4,5,6,7,8,9,10,11,12,13,14,15,16,17]. A conventional ED stack mainly includes cation and anion exchange membranes which were alternatingly set between cathode and anode. Thus, as a key part of the equipment, the ion exchange membrane (IEM) is the research hotspot for the ED process, and their improvement has certainly promoted the development of electrodialysis, along with the progress in operation methods in the industrial field [18,19,20,21]. IEMs include cation exchange membranes (CEMs) and anion exchange membranes (AEMs), which are functionalized with negative and positive ionic groups (i.e., -SO_3_^−^ and -N(CH_3_)_3_^+^), respectively. At present, cation exchange membranes have been successfully commercialized (i.e., Nafion, Neospeta, and Fumapem), but the high cost resulting from the complex preparation of commercial CEMs is unable to be extensively applied in practical production [22,23,24]. Therefore, many researchers direct their efforts towards the preparation of CEMs with high perm-selectivity, good mechanical property and excellent electrochemical stability with acceptable price.

Perfluorosulfonic acid membranes (Dupont Co. Nafion^®^, Wilmington, NC, USA) exhibit low area resistance and excellent chemical stability in the electrodialysis system owing to their phase-separation structure and fluorocarbon frameworks [25]. However, the high cost, low dimensional stability, and serious water transmission result in low current efficiency and limit further application in industry. Su et al. [26] prepared graphene oxide (GO)/Nafion composite membranes with orientated GO nanosheets by a spin-coating method. The ion selectivity was greatly improved and the vanadium-ion permeability of the composite membranes was only 2.64% of the pristine Nafion. However, the spin-coating method determined that it could not prepare a large area of Nafion membrane for industry. Over the past decade, a large number of sulfonated polymers, such as sulfonated polysulfone (PES) [27,28,29], sulfonated poly(ether ether ketone)s (SPEEK) [30], and sulfonated polyvinylidene fluoride (SPVDF) [31], have been developed in order to replace Nafion. These membranes are required to have high ion exchange capacity (IEC) to achieve high ionic conductivity for decreasing energy consumption, which often causes serious swelling behaviors or even dissolution of the membranes in water. Therefore, many researchers directed their efforts towards increasing the conductivity of sulfonated polymer and maintaining a reasonable swelling ratio. Zhou et al. [32] prepared UV-crosslinked sulfonated polysulfone to enhance mechanical properties, to suppress the membrane swelling ratio, and to improve the membrane durability. The water uptake and swelling ratio of these crosslinking membranes decreased by over 50%, but their area resistance increased by about 100%. Farrokhzad et al. [33] made SPVDF and PVDF blend membranes. The water uptake of the membranes was lower than 15% but the ionic conductivity was only 10% of Nafion, which made the membranes unsuitable for practical application due to their high energy consumption. Shukla et al. [34] prepared sulfonated poly(ether ether ketone) (SPK)/imidized graphene oxide (IGO) composite cation exchange membrane. The water uptake of the membranes was effectively suppressed. Moreover, the counterion selectivity and limiting current density was relatively lower than commercial membranes.

Obviously, traditional CEMs cannot make the balance between cost, performance, and service life due to their molecular structure. Therefore, the development of novel cation exchange membranes for electrodialysis has been investigated extensively in recent years. To address the trade-off relationship between the ion conductivity, selectivity, and dimensional stability of conventional cation exchange membranes for electrodialysis, we propose to improve the dimensional stability and selectivity of the membranes through the intermolecular chain entanglement and the interaction between sulfonic acid ions and heterocycles; moreover, the interaction between heterocyclic structures and sulfonic acid groups is used to promote the enhancement of ion conductivity [35,36,37,38]. Poly(phthalazinone ether ketone) (PPEK) is a kind of high performance material with excellent mechanical and thermal properties due to its developed entanglement and heterocyclic structure, which possess the T_g_ of 265 °C [39]. Sulfonated poly(phthalazinone ether ketone) is a kind of membrane material. The membrane has excellent thermal stability, and good conductivity and ion selectivity for its structure [35]. It can be used in flow batteries, fuel cells, and gas separation [36,38]. However, a systematic study of sulfonated poly(phthalazinone ether ketone) for electrodialysis had not yet been undertaken. In this work, sulfonation modification for PPEK was conducted and its molecular structure was characterized. The sulfonated poly(phthalazinone ether ketone) with different ion exchange capacities membranes were prepared, and the electronical-chemical performance was evaluated. An electrodialysis test was carried out for selected membranes to confirm their feasibility of practical electrodialysis application compared with commercial cation exchange membrane.

## 2. Materials and Methods

### 2.1. Materials

Poly(phthalazinone ether ketone) (PPEK, η = 0.81 dL·g^−1^) was dried under a vacuum at 120 °C for 24 h before utilization. The vacuum was supplied by Dalian Baoli New Materials Co., Ltd., (Dalian, China). Chlorosulfonic acid (99.0%) was purchased from Energy Chemical Co., Ltd., (Shanghai, China). The commercial AEMs (TWEDA1S) and CEMs (TWEDC1S) were obtained from Shandong Tianwei Membrane Technology Co., Ltd., (Weifang, China). Other chemicals, such as sodium chloride (NaCl), sodium sulfate (Na_2_SO_4_), *N*-Methyl pyrrolidone (NMP), and concentrated sulfuric acid, were of analytical purity and used without further purification. Deionized water was used throughout all experiments. Deuterated dimethyl sulfoxide (DMSO-d6) and deuterated chloroform (CDCl_3_) was supplied by Energy Chemical Co., Ltd.

### 2.2. Preparation of SPPEK

**Figure 1:**
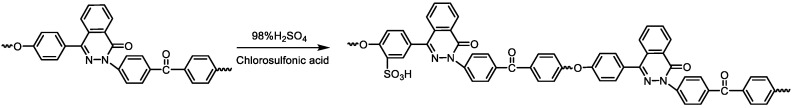
Reaction scheme for the synthesis of SPPEK.

The sulfonated poly(phthalazinone ether ketone) (SPPEK) was obtained by an electrophilic substitution reaction from previous reported works [37,40], as shown in Figure 1. Firstly, 5.0 g PPEK was dissolved in 70 mL concentrated sulfuric acid with mechanical stirring. Then, 10 mL chlorosulfonic acid was slowly added into the mixture under vigorous agitation for 1 h. The reaction was carried out at 90 °C for 2 h and the polymer solution was poured into deionized water with continuous stirring. Finally, the precipitate was washed with deionized water several times until the wasted water was neutral. The product was labeled as SPPEK-1 and dried at 80 °C. Other polymers, such as SPPEK-2, SPPEK-3, and SPPEK-4, were synthesized by controlling reaction time of 3, 5, and 7 h, respectively.

### 2.3. Membrane Preparation

The SPPEK membranes were fabricated by the solution casting method [41]. Take the preparation of SPPEK-1 membrane, for example. A homogenous solution was prepared from 10 wt.% solutions of SPPEK-1 dissolved in NMP. The solution was uniformly poured on to a clean and slick glass plate and evaporated at 60 °C for 4 h. Then, the glass was immersed into deionized water and membranes stripped from glass plate. The membranes were immersed in deionized water. Before the performance examination, an ion exchange was conducted through the membranes with NaCl solution of 0.5 mol·L^−1^ for 24 h.

### 2.4. Characterization

#### 2.4.1. Polymer Characterization

The Hydrogen Nuclear Magnetic Resonance (^1^H-NMR spectra) of PPEK and prepared SPPEKs were measured by a Bruker AVANCE III spectrometer (500 MHz). CDCl_3_ and DMSO-d_6_ were employed as solvents, respectively. The sulfonation degree (*DS*), which reflects the average number of sulfonic acid groups on each repeating unit, was specially calculated by Equation (1) as following [42]:

(1) $$DS=\frac{h_{s}}{h_{a}},$$

where *DS* was the degree of sulfonation, *h_s_* was the integral area of the characteristic peak of proton nearing sulfonic groups, and *h_a_* was the integral area of H-8 proton (peri-proton of carbonyl).

The transformation of *DS* to ion exchange capacity (*IEC*) was calculated by Equation (2):

(2) $$IEC=\frac{1000\times DS}{M+80\times DS},$$

where *M* was molecular weight of repeated unit of PPEK.

The Fourier transform infrared (FT-IR) analysis of products was conducted on a Nicolet 6700 FTIR spectrometer with a method of attenuated total reflection and a total spectral range of 600–4000 cm^−1^.

#### 2.4.2. Thermal Stability of Polymer

The thermogravimetric analysis (TGA) was conducted on a Mettler TGA/SDTA851 instrument from 25 to 800 °C under nitrogen, with a heating rate of 10 °C min^−1^.

#### 2.4.3. Ion Exchange Capacity (*IEC*)

The membrane samples were equilibrated in 1.0 mol·L^−1^ NaCl for 24 h to ensure that -SO_3_H was completely converted to -SO_3_Na. The *IEC* of the membranes was evaluated by titrating soaking NaCl solution with standard NaOH solution using phenolphthalein as an indicator [10]. The ion exchange capacity was calculated by following Equation (3):

(3) $$IEC=\frac{V_{NaOH}\times C_{NaOH}}{m},$$

where *V_NaOH_* was the volume of consumed NaOH solution; *C_NaOH_* was the concentration of NaOH solution; and *m* was the weight of dry membrane, which was produced by drying the membrane at 60 °C for 48 h.

#### 2.4.4. Water Uptake and Swelling Rate

The membranes were immersed in deionized water at 25 °C for 24 h to ensure complete swelling. Then, excess water on the surface was removed by filter paper. The weights and lengths of samples were noted carefully and quickly [38]. Afterward, the membrane samples were dried at 100 °C under vacuum for 24 h. Their weights and lengths were accurately recorded. Water uptake and swelling rate were calculated by the following Equations (4) and (5):

(4) $$WU=\frac{m_{wet}-m_{dry}}{m_{dry}}\times 100\%,$$

(5) $$SR=\frac{l_{wet}-l_{dry}}{l_{dry}}\times 100\%,$$

where *m_dry_* and *l_dry_* were dry weight and length of the membrane; *m_wet_* and *l_wet_* were wet weight and length of the membrane.

#### 2.4.5. Mechanical Properties

The mechanical properties of membranes were measured out by INSTRON 5567A instrument. The membrane samples were prepared into 0.6 × 4 cm rectangles and completely dried, before being tested at a stretch rate of 2 cm·min^−1^ [11].

#### 2.4.6. Scanning Electron Microscopy (SEM)

The membranes were treated with liquid nitrogen for brittle fracture. Cross-section morphologies of the SPPEK membranes were observed by a field-emission SEM 8200 (Hitachi Ltd., Tokyo, Japan).

#### 2.4.7. Membrane Area Resistance

**Figure 2:**
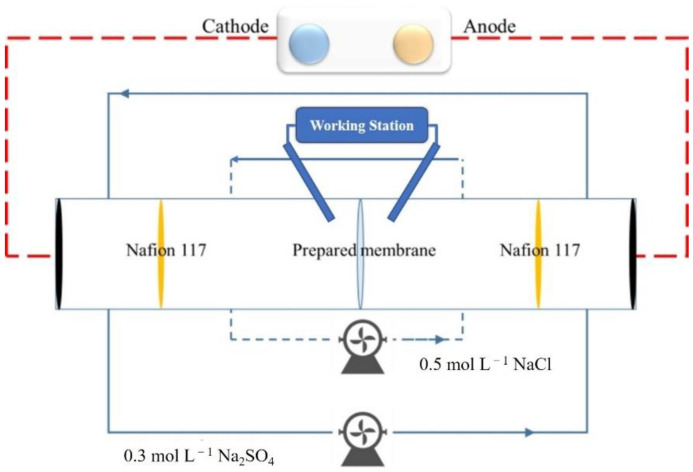
The schematic principle of area resistance measurement.

The area resistance of the SPPEK membranes was measured by a commercial stack obtained from Shandong Tianwei Membrane Technology Co., Ltd. under constant current mode. The instrument used in the process was depicted in Figure 2. The unit consists of two electrode chambers which were separated by two pieces of Nafion 117, respectively, and two intermediate chambers. Intermediate cells were equipped with two reference electrodes (Ag/AgCl) obtained from Shandong Tianwei Membrane Technology Co., Ltd., which were used to measure the potential difference between two sides of tested membrane [43,44]. During the experiment, electrode compartments were fed by 0.3 mol·L^−1^ Na_2_SO_4_ solution and intermediate cells were fed by 0.5 mol·L^−1^ NaCl solution with identical flow rate of 60 mL·min^−1^. A constant current of 0.05 A was supplied by the direct current power source (GPS-X303/C, Good Will Instrument Co., Ltd., Shanghai, China), and the potential between two electrodes was recorded by an electrochemical station (Zennium E4, ZAHNER-elektrik GmbH & Co., Kronach, Germany). The membrane area resistance was calculated by Equation (6) given below:

(6) $$R=\frac{U-U_{0}}{I}\times S,$$

where, *R* was area resistance of tested membranes; *U* and *U*_0_ were potential of experiment stack with and without membranes; *I* was constant current of 0.05 A; *S* was effective membrane area (7 cm^2^).

#### 2.4.8. Transport Number

The instrument, shown in Figure 2, was also used to evaluate transport number without external current and feeding electrode solution into electrode chambers at 25 °C. According to the reported method [42,43], 0.5 mol·L^−1^ NaCl solution and 0.1 mol·L^−1^ NaCl solution were pumped into intermediate cells, respectively. The potential between two sides of membrane was measured by reference electrode. The transport number was calculated by Equation (7) listed below:

(7) $$\tau =\frac{E_{m}+E_{0}}{2E_{0}},$$

where *E_m_* was trans-membrane potential and *E*_0_ was standard potential between two feeding solution.

#### 2.4.9. Electrodialysis Experiment

**Figure 3:**
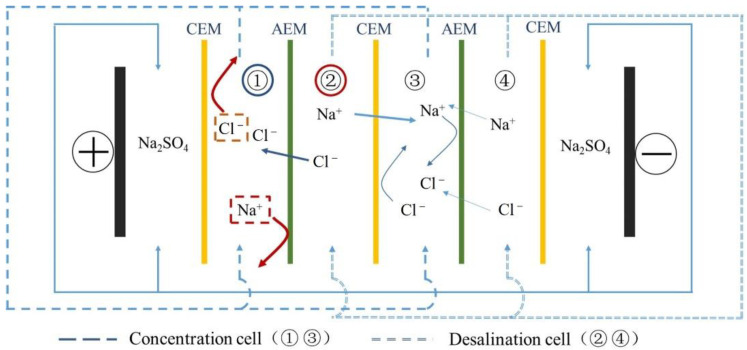
The schematic principle of ED stack.

The desalination performance of the membranes was evaluated by electrodialysis. The ED stack was purchased from Shandong Tianwei membrane Technology Co., Ltd. (Weifang, China). The stack of electrodialysis consisted of alternating CEM and AEM (3 CEMs and 2 AEMs) with an effective area of 7 cm^2^, and the electrodes were titanium electrodes coated with ruthenium, as demonstrated in Figure 3. TWEDA1S was used as AEM. Electrode chambers were fed with 0.3 mol·L^−1^ Na_2_SO_4_ solution (250 mL) and were connected to avoid pH fluctuation. Diluted chambers and concentrated chambers were both fed with 0.1 mol·L^−1^ NaCl solution (250 mL) [43]. Before ED process, the stack was circulated for 30 min in order to remove visible bubbles. Next, a constant current of 0.14 A was supplied by direct current power source (GPS-X303/C, Good Will Instrument Co., Ltd., Shanghai, China). The experiment was conducted for 180 min. The change of conductivity in the concentrated cell and the diluted cell was recorded by a conductivity meter (DDS-307, INESA Scientific Instrument Co., Ltd., Shanghai, China) during the test every 15 min. To compare desalination performance, the ED experiment of commercial CEM TWEDC1S (polystyrene cation exchange membrane, labeled as CMX in the following) was carried out under the same conditions. The desalination and concentration rates, current efficiency, and energy consumption were calculated as following Equations (8)–(11) [31,32]:

(8) $$R_{d}=\frac{C_{0}-C_{t}}{C_{0}},$$

(9) $$R_{c}=\frac{C\prime_{t}-C\prime_{0}}{C\prime_{0}},$$

(10) $$CE=\frac{F\left(C_{0}-C_{t}\right)V_{0}}{NIt},$$

(11) $$E=\int_{0}^{t}\frac{UI}{\left(C_{0}V_{0}-C_{t}V_{t}\right)M}\mathrm{dt},$$

where *C*_0_ and *C_t_* were initial conductivity and conductivity at t min in desalination cell (DC), *C*′_0_ and *C*′*_t_* were initial conductivity and conductivity at t min in concentration cell (CC), *F* was Faraday constant; *I* was current of 0.14 A, *U* was applied voltage, *M* was molecular weight of NaCl, *N* was the number of ED module unit, *t* was consumed time and *V*_0_ and *V_t_* were initial volume and volume at *t* of NaCl solution.

#### 2.4.10. Current–Voltage Curves Measurement

The current–voltage (I-V) curves of the membranes were measured using the same unit mentioned above. The tested cell was recirculated with 0.5 mol·L^−1^ NaCl at a flow rate of 60 mL·min^−1^, and 0.5 mol·L^−1^ Na_2_SO_4_ was used as an electrode rinse solution and circulated at the same flow rate [45]. For I-V curves measurement, a stepwise current was applied by direct current power source (GPS-X303/C, Good Will Instrument Co., Ltd.) and the corresponding potential difference across the membrane was recorded from the direct current power source.

#### 2.4.11. Saline Water Treatment

The operation of saline water concentration was similar to standard electrodialysis measurement. The electrodialysis device used for high concentration brine treatment is the same as the standard electrodialysis device. Electrode chambers were fed with 0.3 mol·L^−1^ Na_2_SO_4_ solution (250 mL). The diluted cell and concentrated cell were each fed with higher concentration 50 mL NaCl solution (0.6 mol·L^−1^), and the operation current was 0.18 A.

## 3. Results and Discussion

### 3.1. Synthesis of SPPEK and Chemical Structure Characterization

**Figure 4:**
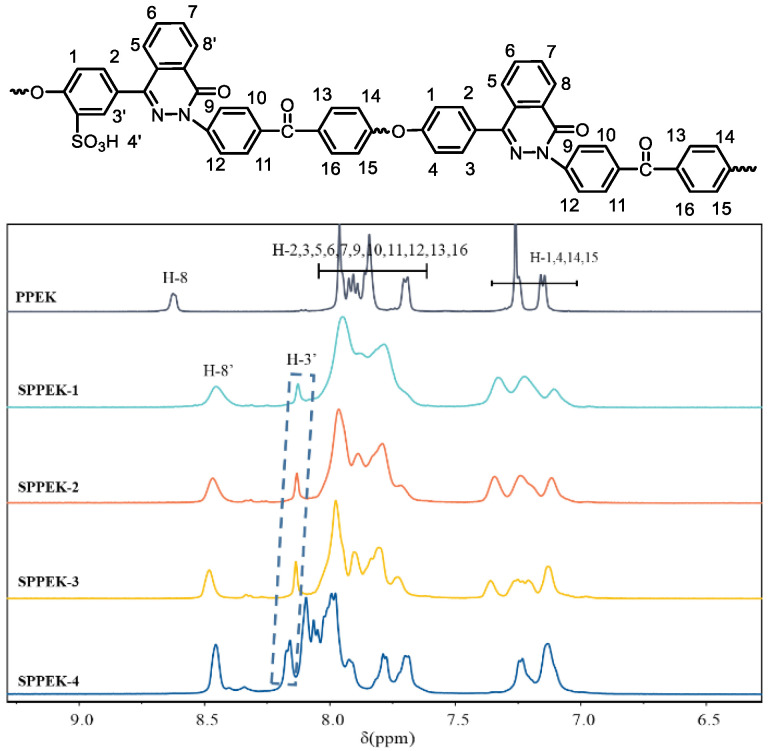
^1^H-NMR spectra of PPEK and SPPEK.

The chemical reaction of SPPEK was illustrated in Figure 1. The chlorosulfonic acid was able to substitute hydrogen the atom which was near the ether bond. By controlling the reaction time, SPPEKs with different *IEC* were prepared. The reaction conditions of SPPEKs and their *IECs* were exhibited in Table 1. By increasing the reaction time from 2 to 7 h, the *IEC* of SPPEK increased from 0.77 to 1.82 mmol·g^−1^. The IECs characterized by titration were consistent with the results of ^1^H-NMR spectra in Figure 4 (normalized the area of H-8 peak and calculated the peak of H-3′ signal).

**Figure 5:**
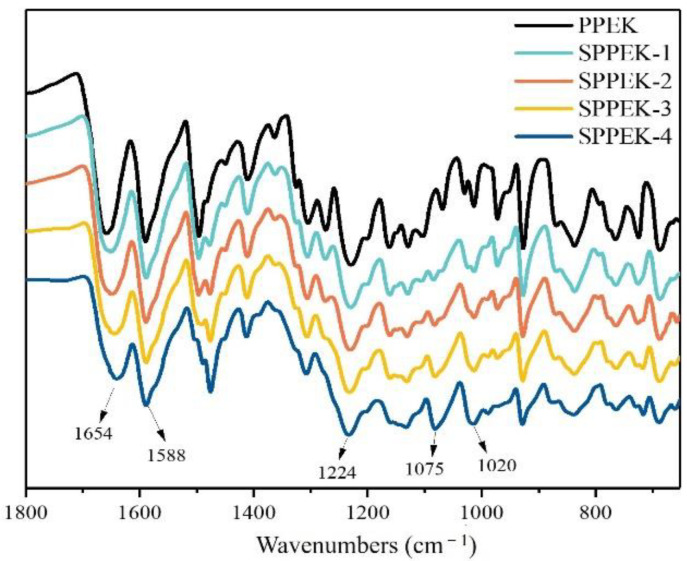
FT-IR spectra of PPEK and SPPEK.

The chemical structures of SPPEKs were confirmed by ^1^H-NMR and FT-IR, which are presented in Figure 4 and Figure 5. As shown in Figure 4, the new proton signals found around 8.12–8.20 ppm were protons nearing sulfonate groups. This is attributable to electron withdrawing effect of adjacent -SO_3_H and the signals moved to higher chemical shift region. The difference in this characteristic peak was ascribed to the content of sulfonic groups. In addition, the integral area of characteristic signal could be used to calculate ion exchange capacity, the values of which were listed and compared with the titration method in Table 1. The ion exchange capacities calculated from the ^1^H-NMR spectra were in general agreement with the titration values. The initial signal at 8.62 ppm of PPEK shifted to around 8.45 ppm, which was due to the different deuterated solvents (PPEK with CDCl_3_ and SPPEKs with DMSO-d_6_). Other proton signals were assigned to according groups.

As shown in Figure 5, the characteristic bands at 1654 cm^−1^ and 1588 cm^−1^ were assigned to stretching vibration of C=O and benzene rings in polymer backbone, respectively. Compared with the spectroscopy of PPEK, the new absorption peaks at 1020 cm^−1^ and 1075 cm^−1^ found in spectrum of SPPEK were symmetric and asymmetric stretching vibrations of aromatic -SO_3_H, respectively [35]. Moreover, the intensity of absorption peaks increased with an increase of IEC, which confirmed that sulfonic groups were successfully grafted onto the PPEK chemical structure.

### 3.2. Thermal Analysis

**Figure 6:**
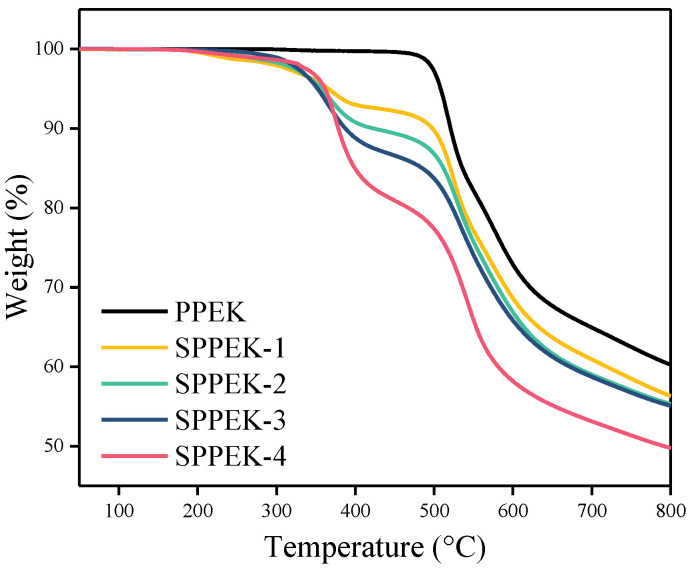
TGA curves of PPEK and SPPEK with different IECs.

The thermal stability of PPEK and SPPEK were measured by thermogravimetric analysis (TGA). As shown in Figure 6, PPEK exhibited a main degradation process while all SPPEKs had two stages of weight loss. The first degradation stage of SPPEKs appeared from 260 to 400 °C, which was attributed to the degradation of sulfonate groups [9,41]. During this stage, the weight loss of SPPEKs were about 7.3, 9.6, 12.0, and 16.4%, respectively, which was approximately equal to the weight of sulfonate groups, indicating the completely degradation of functional groups. A 5% weight loss of SPPEKs was about 360 °C, suggesting their good thermal stability. The last degradation region of SPPEK samples and PPEK presented at 500 °C, which was recognized as the decomposition of polymer main chain [9,41].

### 3.3. Morphology and Mechanical Properties of the Membranes

**Figure 7:**
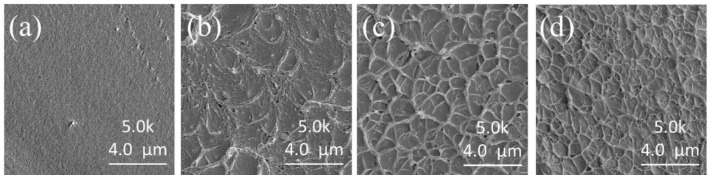
Cross-section microstructure of membranes, (**a**) SPPEK-1, (**b**) SPPEK-2, (**c**) SPPEK-3, (**d**) SPPEK-4.

As shown in Figure 7, the cross-section image of SPPEK-1 was dense and smooth, but SPPEK-2 had some grid structure. With the increase of IEC, their morphologies were getting rougher, which resulted from the interaction of sulfonic groups [38]. Mechanical properties of SPPEK membranes, including tensile strength and elongation at break, were measured, and their results were shown in Table 2. The tensile strength of CEMs was found to decrease from 84.0 to 68.5 MPa with an increase in IEC. The mechanical performance of the membranes was eroded with IEC improvement. This resulted from the sulfonic groups in SPPEK membranes inducing phase separation [38], and the rougher microstructure damaged the uniformity of membranes in Figure 7 and decreased tensile strength [38]. The SPPEK-4 possessed the lowest tensile strength (68.5 MPa) but the highest elongation at break (63.6%), which could avoid breaking during ED process. Herein, the mechanical properties being experimented indicated that the SPPEK membranes can be applied in the electrodialysis process and other electrochemical applications.

### 3.4. Electrochemical Properties of the Membranes

The content of sulfonic groups played an important role in ion transport, which directly influenced the efficiency of ion migration and energy consumption in the ED process. As shown in Table 3, the area resistance greatly decreased from 51.29 to 0.62 Ω·cm^2^ with the IEC increasing from 0.77 to 1.82 mmol·g^−1^, which was due to higher contents of ion exchange groups, with wider and continuous ion transport channels. In addition, the increase of the IEC also improved ion selectivity, which was due to strong Donnan effects of ion exchange groups (-SO_3_^−^) [38,46], resulting in a weak transmembrane transport of anions such as Cl^-^, so the transport number increased from 0.90 to 0.97. However, compared with SPPEK-3 (IEC = 1.45 mmol·g^−1^) and SPPEK-4 (IEC = 1.82 mmol·g^−1^), the ion selective transport property did not enhance, which was attributed to high water uptake and swelling rate of SPPEK-4 [38,46].

### 3.5. Limiting Current Density of the Membrane

**Figure 8:**
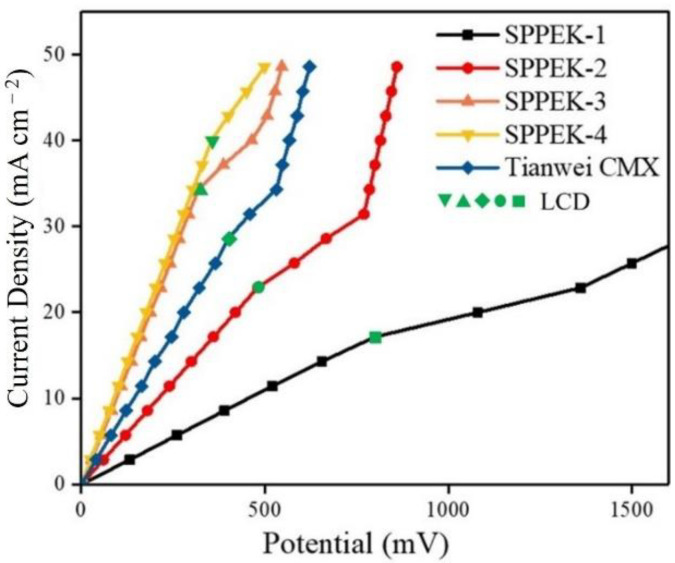
I-V curves of SPPEK membranes and commercial for comparison.

Limiting current density (LCD) was an important parameter in the practical ED process. The ED process involves a boundary between membranes and solution. Ions should migrate across the boundary and transport across membranes. In general, the transport of ions across membranes was faster than that across the boundary between membrane and stream [45,47]. Ion migration velocity increased with an increase of applied current, and the counterions stayed in the boundary. The number of counterions leaving the boundary layer was equal to the number of transmembrane conduction ions under a low current density, and the ions in the boundary showed an equilibrium diffusion process. Meanwhile, the number of transmembrane conduction ions was more than the number of counterions, and a counter potential was formed in the boundary under the excessive current density, resulting in an increase in mass transfer resistance and a decrease in current efficiency, which was called a polarization. The current density corresponding to the transition of the ion conduction process within the boundary layer from the equilibrium diffusion state to the polarization state was defined as the limiting current density. In practical ED operation, the applied current was commonly lower than LCD in order to protect equipment and avoid unnecessary energy consumption resulting from the electrolysis of water. For investigating the LCD of novel membranes, current-voltage curves were measured with linear sweep voltammetry (LSV) method and shown in Figure 8. A traditional curve of the tested sample exhibited three typical stages. Initially, the potential increased linearly with the current density, which was known as an ohmic region due to the balance between boundary and membrane surface. Next, a flat stage appeared when the current increased to a certain value as a result of the counter potential generated by the concentration difference between membrane surface and solution boundary. The LCD was commonly defined as the intersection of ohmic region and flat stage. Finally, the current rapidly increased following a potential slight increase. Table 4 listed the precise LCD value of all membranes. LCD exhibited a positive correlation with ion exchange capacity; SPPEK-4 membrane, which had the highest IEC, had an LCD up to 39.8 mA·cm^2^, which was superior to other SPPEK membranes and commercial CMX membrane (28.3 mA·cm^2^). Obviously, the content of sulfonic groups could explain this result. The hydrophilic groups in membranes tightly capture water so as to increase the mixture of solution and boundary, which enhances the mass transfer across boundary and achieved higher balance between membrane surface and this boundary [45,47]. Therefore, SPPEK-4 had excellent LCD performance.

### 3.6. Electrodialysis Experiment

**Figure 9:**
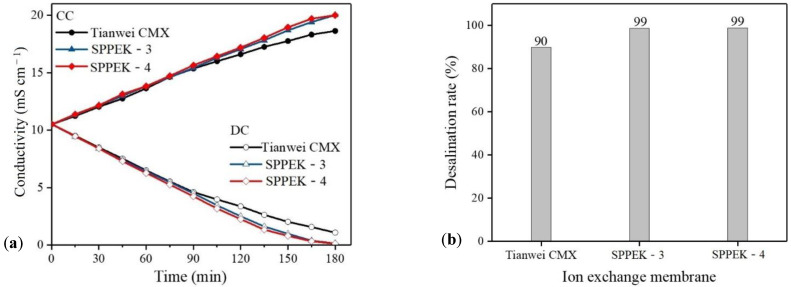

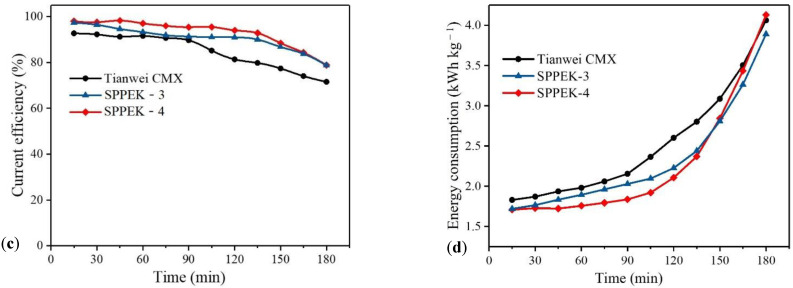
The ED performance of SPPEK-3, SPPEK-4, and CMX membranes; (**a**) ion conductivity of standard solution in DC and CC; (**b**) the final salt removal rate; (**c**) current efficiency; (**d**) energy consumption of ED equipment.

SPPEK-3 and SPPEK-4 membranes were fabricated under the optimal conditions and their ED performance was evaluated in a laboratory-scale stack operated under the constant current method, along with commercial membrane for comparison. As shown in Figure 9a, during the ED process, the desalination and concentration performance of different membranes shared a similar trend: in the beginning stage, a linear relationship appeared between conductivity of solution and time, before the rate of change of conductivity became gradually smaller with time due to the polarization phenomenon between DC and CC after ED operation for 90 min. The result was consistent with their current efficiency experiment in Figure 9b. The current efficiency of the SPPEK-4 membrane was close to 97% at first, and then decreased to 79% as the time of this process increased, while the CMX membrane performed the initial current efficiency of 92% and the current efficiency gradually reduced to 71% at the end of test. Furthermore, the SPPEK-4 membrane exhibited the highest salt removal rate of 99%, which was superior to the commercial membrane (desalination rate of 90%). All of the above results were due to the favorable cation conduction performance and ion selectivity of the SPPEK-4 membrane, which possesses the highest ion transport number [42]. In addition, the energy consumption of all membranes initially changed slightly and then rapidly increased after 120 min due to current carrier reduction in DC. In the beginning stage, the energy consumption for SPPEK-3 and SPPEK-4 membranes (1.72 kWh·kg^−1^) was lower than the commercial membrane, CMX (1.83 kWh·kg^−1^), which was caused by the low resistance of SPPEK-3 and SPPEK-4 membranes. However, after 150 min, the energy consumption of SPPEK-3 and SPPEK-4 membranes increased rapidly, even exceeding the CMX membrane, which resulted from the much lower conductivity (salt concentration) in DC. Thus, considering their excellent desalination performance and energy consumption, the SPPEK-4 and SPPEK-3 membranes can still be expected to be applied in practical electrodialysis.

### 3.7. Saline Water Treatment

**Figure 10:**
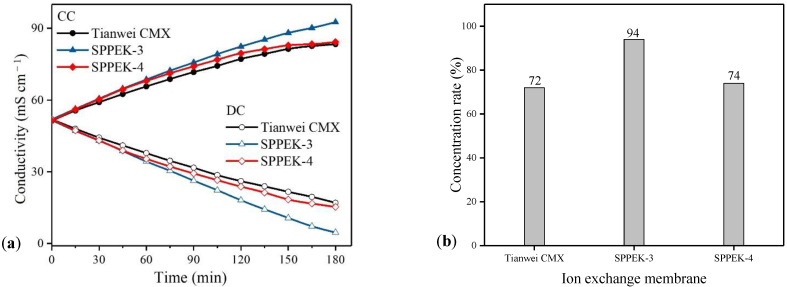

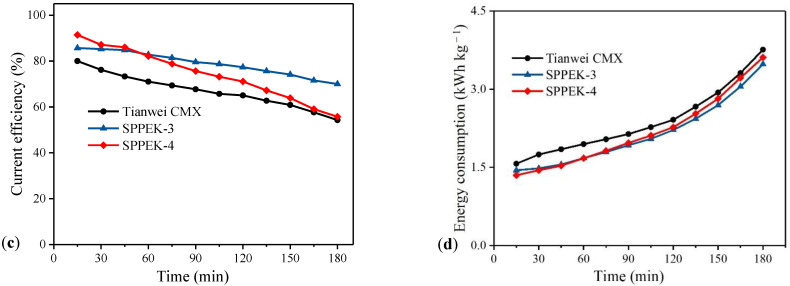
The ED performance of SPPEK-3, SPPEK-4 and CMX membranes for saline water, (**a**) ion conductivity of saline solution in DC and CC; (**b**) the final salt removal rate; (**c**) current efficiency; (**d**) energy consumption of ED equipment.

ED can treat the effluent of concentrated brine from industrial applications of reverse osmosis (RO) for avoiding damage to marine ecosystems. As shown in similar standard solution, the ion conductivity of feeding streams in CC increased, while conductivities in DC decreased. Initially, the solution concentrations in CC were similar between SPPEK-3 and SPPEK-4. However, SPPEK-3 membrane showed better performance after 75 min. The final desalination and concentration rates of SPPEK-3 were 94% within 180 min, while 72% for SPPEK-4 membranes in Figure 10b. The results in this study were attributed to a higher back diffusion of counterions appearing in the SPPEK-4 membrane, which resulted from this membrane possessing a higher water-swelling behavior than the SPPEK-3 membrane [42]. During the ED process, water transport was in line with ion migration. The membrane with lower water uptake easily allowed slight water transportation under concentration difference and external potential [34,46]. Therefore, the higher water transport of SPPEK-4 negatively influenced its performance for treating saline water. In addition, the energy consumption and current efficiency were 3.59 kWh·kg^−1^ and 68%, respectively, for the SPPEK-3 membrane, which was 34% higher in current efficiency and 7% lower in energy consumption compared to the CMX membrane. In conclusion, the SPPEK-3 membrane had the best properties for treating high salinity wastewater.

## 4. Conclusions

A series of sulfonated poly(phthalazinone ether ketone) (SPPEKs) with different IEC (0.77 to 1.82 mmol·g^−1^) were synthesized via electrophilic substitution reaction by chlorosulfonic acid. The ^1^H-NMR and FT-IR analysis confirmed successful insertion of sulfonate groups. TGA curves showed that the degradation temperature of SPPEKs was approximately 290 °C, indicating the excellent thermal stability of SPPEKs. The mechanical properties of SPPEK membranes decreased with the addition of sulfonic groups, but maintained relatively good mechanical properties (over 68.5 MPa) and outstanding elongation at break (63.6%). Compared with CMX, SPPEK-4 had higher ion selectivity (0.97) and limiting current density (39.8 mA·cm^2^) and lower area resistance (0.62 Ω·cm^2^), which demonstrated excellent electrochemical properties. The possibility of the practical applications of membranes was evaluated by ED process. The results showed that the SPPEK-4 had a higher salt removal rate of 99% than the CMX, indicating its potential in ED application. Additionally, the SPPEK-3 membrane showed outstanding performance in concentration rate (94%) and low energy consumption (4.33 kWh·kg^−1^), which indicated the feasibility of novel membranes in ED application.

## Figures and Tables

**Table 1:** The conditions and results of sulfonation reaction.

Sulfonated Polymer	PPEK (g)	Chlorosulfonic Acid (mL)	Reaction Time (h)	*IEC* ^a^ (mmol·g^−1^)	*IEC* ^b^ (mmol·g^−1^)
SPPEK-1	5	10	2	0.77	0.76
SPPEK-2	5	10	3	1.02	1.04
SPPEK-3	5	10	5	1.45	1.40
SPPEK-4	5	10	7	1.82	1.88

^a^*IEC* was calculated by titration; ^b^ *IEC* was calculated by ^1^H-NMR spectra.

**Table 2:** Mechanical properties of SPPEK membranes.

Membranes	Tensile Strength (MPa)	Elongation at Break (%)
SPPEK-1	84.9	18.2
SPPEK-2	80.8	30.0
SPPEK-3	78.7	52.6
SPPEK-4	68.5	63.6

**Table 3:** The electrochemical properties and dimensional stability of prepared membranes and commercial CMX.

Membranes	IEC (mmol·g^−1^)	Area Resistance (Ω·cm^2^)	Transport Number	Water Uptake (%)	Swelling Rate (%)
SPPEK-1	0.77	51.29 ± 0.27	0.90	4.7 ± 0.8	2.9 ± 0.1
SPPEK-2	1.02	15.41 ± 0.23	0.93	9.0 ± 0.9	4.1 ± 0.3
SPPEK-3	1.45	1.13 ± 0.18	0.97	23.0 ± 0.8	7.3 ± 0.8
SPPEK-4	1.82	0.62 ± 0.10	0.97	40.2 ± 1.3	10.4 ± 1.3
CMX	1.08	2.35 ± 0.13	0.93	18.4 ± 2.3	6.4 ± 0.6

**Table 4:** Characteristic values from I–V curves.

Membranes	LCD (mA·cm^2^)	R_ohm_^a^ (Ω·cm^2^)
SPPEK-1	17.0	6.75
SPPEK-2	23.2	3.03
SPPEK-3	34.3	1.35
SPPEK-4	39.8	1.23
CMX	28.3	2.06

^a^ the resistance of ohmic region.

## Data Availability

The data presented in this study are available on request from the corresponding author.
